# PTD-mediated delivery of α-globin chain into Κ-562 erythroleukemia cells and α-thalassemic (HBH) patients’ RBCs ex vivo in the frame of Protein Replacement Therapy

**DOI:** 10.1186/s40709-021-00148-3

**Published:** 2021-07-20

**Authors:** Androulla N. Miliotou, Dionysia Papagiannopoulou, Efthymia Vlachaki, Martina Samiotaki, Dimitra Laspa, Stamatia Theodoridou, Asterios S. Tsiftsoglou, Lefkothea C. Papadopoulou

**Affiliations:** 1 Laboratory of Pharmacology, Department of Pharmacognosy - Pharmacology, School of Pharmacy, Faculty of Health Sciences, Aristotle University of Thessaloniki, 54124 Thessaloniki, Macedonia Greece; 2 Department of Pharmaceutical Chemistry, School of Pharmacy, Aristotle University of Thessaloniki, 54124 Thessaloniki, Macedonia Greece; 3 Adult Thalassemia Unit, Hippokrateion General Hospital, 54642 Thessaloniki, Macedonia Greece; 4 Institute for Bioinnovation, Biomedical Sciences Research Centre “Alexander Fleming”, 16672 Vari, Greece

**Keywords:** Intracellular transduction via PTD, TAT-α-globin, Size exclusion chromatography, LC − MS/MS analysis, HbH thalassemic patients’ RBCs

## Abstract

**Background:**

α-Thalassemia, a congenital hemoglobinopathy, is characterized by deficiency and/or reduced levels of α-globin chains in serious forms of α-thalassemia (HbH disease/Hb Bart’s). This research work deals with a Protein Replacement Therapy approach in order to manage α-thalassemia manifestations, caused by the excess of β-globin chain into HbH RBCs. The main goal was to produce the recombinant human α-globin chain in fusion with TAT, a Protein Transduction Domain, to ex vivo deliver it into HbH patients RBCs, to replace the endogenous missing α-globin chain.

**Results:**

Cloning of the α-globin coding sequence, fused to the nucleotide sequence of TAT peptide was conducted and the human recombinant fusion proteins, 10xHis-Xa_SITE_-α-globin-HA and 10xHis-Xa_SITE_-TAT-α-globin-HA were produced. The ability of human recombinant 10xHis-Xa_SITE_-α-globin-HA to interact in vitro with the previously produced 10xHis-Xa_SITE_-TAT-β-globin-HA and form α-/β-globin heterodimers, was assessed and confirmed by size exclusion chromatography. The recombinant 10xHis-Xa_SITE_-TAT-α-globin-HA was successfully delivered into human proerythroid K-562 cells, during the preliminary transduction evaluation experiments. Finally, the recombinant, TAT-fused α-globin was successfully transduced into RBCs, derived from HbH patients and reduced the formation of HbH-Inclusion Bodies, known to contain harmful β_4_-globin chain tetramers.

**Conclusions:**

Our data confirm the successful ex vivo transduction of recombinant α-globin chains in HbH RBCs to replace the missing a-globin chain and reduce the HbH-inclusion bodies, seen in α-thalassemias. These findings broaden the possibility of applying a Protein Replacement Therapy approach to module sever forms of α-thalassemia, using recombinant α-globin chains, through PTD technology.

**Supplementary Information:**

The online version contains supplementary material available at 10.1186/s40709-021-00148-3.

## Background

Protein Replacement Therapy (PRT) confers an alternative therapeutic approach to monogenic disorders, where a mutated gene encodes either an abnormal protein or no protein at all. Recombinant therapeutic proteins administered as medicinal agents into the body to function either in plasma or via binding to cell surface receptors or even intracellularly, in case they can penetrate the cell membrane natural selective barrier [1]. To transduce biomembranes, biologics must be either highly lipophilic or of very small size or there must be transported via a specific uptake mechanism. Thus, due to this inability to cross the cellular membrane, efficient intracellular delivery of recombinant protein therapeutics remains problematic. Thanks to the Protein Transduction Domain (PTD) technology, developed with the use of Protein Transduction Domains (PTDs) or Cell Penetrating Peptides (CPPs) that facilitate transduction, different heterologous cargos, large or small bioactive molecules have been delivered successfully into nearly all eukaryotic cells [2–12].

TAT peptide, as the first PTD derived from HIV TAT protein sequence [13, 14], has attracted worldwide attention as suitable for efficient intracellular transduction of cargo. With safety and efficacy being still major issues for wider application of gene therapy of monogenic disorders, PTD technology for protein delivery in the frame of PRT has been considered an alternative approach, without interfering with the host’s genome [15], and there are many ongoing clinical trials [2, 6] towards this goal.

Over the years, our group has been engaged in a series of studies using the PTD mediated protein delivery in human cultured cells. These include the successful delivery of human recombinant mitochondrial TAT-L-Sco2 fusion protein into the mitochondria of a cytochrome c oxidase (COX) deficient cell culture model bearing *SCO2* mutations [1, 16, 17], the in vivo biodistribution of the radiolabeled recombinant TAT-L-Sco2 protein in mice [18] and the transduction of the human recombinant TAT-β‑globin chain into proerythroid K‑562 cells to replace the missing β‑globin as a therapeutic approach to β-thalassemia. The latter study confirmed the formation of hemoglobin α_2_β_2_-like tetramer, although to a limited extent [19]. The long-term goal of these studies has been to establish the PTD-mediated recombinant protein delivery as an alternative protein therapeutic approach for monogenetic disorders, including the β-thalassemias.

Thalassemias are caused by mutations or deletions in human globin genes and result in the deficiency or reduced levels of functional hemoglobins in red blood cells (RBCs). Alpha (α-) thalassemia is caused by, mainly, deletions among the four α-globin genes, and/or point mutations of α-globin genes (HBA2 and HBA1); approximately 293 deletional or non-deletional variants have been identified [20, 21]. The imbalance between α- and β- chains affects the severity of α-thalassemias, which is well correlated with the number of functional copies of the α-globin genes. This leads to various clinical phenotypes, ranging from asymptomatic to thalassemia intermedia Hemoglobin H (HbH) disease and lethal Hb Bart's hydrops fetalis (BHFS). In BHFS, hemoglobin tetramers of only γ-chains (γ_4_) are ineffective in erythropoiesis and oxygen delivery to tissues and small amounts of Hb Portland I and Portland II support the survival of the fetus at the final stage of pregnancy [22–24]. Increasing numbers of BHFS in newborns have been reported worldwide. For the last decades, the survival of patients with BHFS is possible, due to intrauterine blood transfusions, but not without short and long-term complications [23] and continuous lifelong transfusions (early or late systematical transfusions) in order to be alive.

As there is no other therapy, except red blood cell transfusions, new therapeutic approaches for the serious forms of α-thalassemia (HbH disease / Hb Bart’s) are required. The production and delivery of the α-globin gene translational product itself may be considered as a potential treatment of α-thalassemia. This study aimed to: (i) produce the fusion recombinant 10xHis-Xa_SITE_-α-globin-HA and 10xHis-Xa_SITE_-TAT-α-globin-HA proteins, (ii) assess the in vitro formation of a stable complex of the 10xHis-Xa_SITE_-α-globin-HA with the corresponding 10xHis-Xa_SITE_-TAT-β-globin-HA and (iii) transduce the 10xHis-Xa_SITE_-TAT-α-globin-HA deliberately in vitro*,* into K-562 proerythroid cells and ex vivo*,* into RBCs derived from HBH patients, in order to alter the disease’s phenotype, by reducing the formation of the harmful β_4_-tetramers into patients’ RBCs.

## Methods

### Oligonucleotides

All pair of primers (Forward/Backward) used are listed below. Restriction enzyme sites, incorporated to facilitate cloning, are underlined and indicated in parentheses.

F-α: 5΄-ATG GTG CTG TCT CCT GCC GAC-3΄, B-α: 5΄-ACG GTA TTT GGA GGT CAG CAC GGT-3΄, F-TAT-α: 5΄-GCA GGT TCA TAT GCG CAA GAA ACG CCG CCA GCG CCG CCG CAT GGT GCT GTC TCC T-3΄ (*Nde*I), B-α-HA: 5΄-CAA CTC GAG TCA AGC ATA GTC TAA GAC GTC ATA ATA ACG GTA TTT GGA GGT CAG-3΄ (*Xh*oI), F-α2: 5΄- GCA GGT TCA TAT GGT GCT GTC TCC TGC CGA CAA-3΄ (*Nde*I).

### Construction of recombinant vectors

The cloning of the α-globin coding sequence (CDS) fused to the nucleotide sequence of the TAT peptide and the Hemagglutinin (HA) tag was carried out in both pCRII-TOPO vector (TOPO® TA Cloning® Kit with pCRII® TOPO®, Thermo Fisher Scientific, Massachusetts, United States) and the bacterial fusion expression vector pET-16b (Novagen, Darmstadt, Germany) [19].

Using human placenta -DNase treated- total RNA as a template and primer pairs F-α / B-α the α-globin CDS [*Homo sapiens* hemoglobin, alpha 1 (HBA1), mRNA NCBI Reference Sequence: NM_000558.4] was amplified using DreamTaq™ Hot Start DNA Polymerase (Thermo Fisher Scientific, Massachusetts, United States). The PCR product (429 bps) was ligated to pCRII-TOPO vector. Using this plasmid, as a template, and the primer pairs F-TAT-α-ΗΑ/B-α-HA and F-α2/B-α-HA, the TAT-α-globin-HA (485 bps) and α-globin-HA gene fragments (454 bps), respectively, were amplified. The two PCR products developed were then ligated to pCRII-TOPO. These plasmids were then digested and proceeded in «sticky ends» cloning, with the corresponding restriction enzymes, into pET-16b to generate the recombinant plasmids. Freshly prepared competent *E. coli* strain TOP10F' were transformed with the recombinant prokaryotic plasmids and plasmid DNA was isolated (Nucleospin Plasmid Kit, Macherey–Nagel, Düren, Germany). Clones containing the correct construct-inserts were selected via RFLP analysis and verified by automatic sequence analysis (CeMIA SA, Larissa, Greece).

### Expression, purification and analysis of recombinant proteins

*Escherichia coli* strain CD43 (DE3) was transformed by the pET-16b-recombinant prokaryotic plasmids to express 10xHis-Xa_SITE_-α-globin-HA and 10xHis-Xa_SITE_-TAT-α-globin-HA proteins, as previously described [17–19]. The fusion proteins were purified by affinity Ni^2+^-NTA column chromatography in order to be used in the Κ-562 transduction experiments, in the in vitro assembly of α- and β-monomers and in the size exclusion HPLC experiments. For the affinity Ni^2+^-NTA column chromatography, HisPur™ Ni–NTA Resin agarose beads (Thermo Fisher Scientific, Massachusetts, United States) were used and the recombinant proteins were eluted from the column by adding gradient imidazole concertation buffers (containing 10 mM—400 mM imidazole) and then filtrated using a stirred ultrafiltration cell (Millipore, Massachusetts, United States) for purification.

Alternatively, bacterial pellets were collected and processed for isolation and purification of bacterial Inclusion Bodies (bacterial-IBs), dissolved in 1 M l-Arginine (l-Arg) Sigma-Aldrich, St. Louis, Missouri, United States) [17, 19] and further used in the transduction experiments, as in our previous works [17–19].

SDS − PAGE and Western Blot immunostaining analysis were carried out, as previously described [17]. The resolved proteins were separated in 14–15% SDS-PAGE and blotted with the mouse monoclonal anti-His.IgG (1:2600) (Sigma-Aldrich, St. Louis, Missouri, United States), the mouse monoclonal anti-HA-(antihemagglutinin).IgG (Santa Cruz Biotechnology Inc. California, United States) and rabbit polyclonal anti-GAPDH antibody (1:4000) (Flarebio Biotech LLC, New Jersey, United States). The membrane was then incubated with alkaline phosphatase-conjugated goat anti-mouse.IgG-AP (1:1000) (Santa Cruz Biotechnology Inc. California, United States) and goat anti-rabbit.IgG-AP (1:2500) (Sigma-Aldrich, St. Louis, Missouri, United States). Proteins were visualized by using NBT/BCIP (Biotium, California, United States) substrates for alkaline phosphatase.

### LC − MS/MS technology for recombinant protein identification

Bacterial-IBs, enriched in recombinant 10xHis-Xa_SITE_-TAT-α-globin-HA, as well as soluble (purified by Ni^2+^-NTA chromatography) 10xHis-Xa_SITE_-α-globin-HA and 10xHis-Xa_SITE_-TAT-β-globin-HA (used for in vitro assembly/formation into α-/β-globin tetramer analyzed by size exclusion HPLC) were subjected in LC − MS/MS analysis for protein sequence identification (Additional file 1: Methods).

### In vitro assembly of α- and β- monomers and analysis by size exclusion HPLC

The in vitro assembly of α- and β- monomers into α/β-globin tetramers was evaluated when equal quantities of the recombinant 10xHis-Xa_SITE_-α-globin-HA protein (∼40 μg) and of the 10xHis-Xa_SITE_-TAT-β-globin-HA [19] (both, solely, purified by affinity Ni^2+^-NTA column chromatography) were mixed and incubated at R/T (Room Temperature) for different timed-intervals. The analysis of the reaction mixture containing the monomers of the recombinant α- and β-chains was achieved by size exclusion chromatography (SEC) at t = 0, 24 and 48 h post-incubation. Prior to incubation, both the recombinant proteins were incubated with 10 mM DTT for 1 h to reduce globin-dimers in the corresponding sample monomers [25]. Also, soluble recombinant proteins were separately, in different runs, analyzed by LC–MS/MS, as previously described, to identify the monomers’ protein sequence.

The SEC-HPLC system comprised of an Agilent HP 1100 series pump (Agilent, California, United States) multiple wavelength detector set at 270 nm and a Phenomemex BIOSEP-SEC-S3000 column (300 × 7.8 mm, 5 μm, Phenomenex, California, United States). The mobile phase was phosphate buffer 50 mM pH 6.8, containing 0.3 Μ ΝaCl and the flow rate was 0.5 mL min^−1^.

### Cell culture and transduction experiments

Human Chronic Myelogenous Leukemia K-562 cells [26] were cultured in RPMI-1640 medium supplemented with 10% fetal bovine serum (FBS) and antibiotics/antimycotic (Gibco-Invitrogen, Life Technologies Inc., Texas, United States) at 37 °C, in 5% CO_2_. For the transduction of TAT fusion protein, the cells were cultured to 70–80% confluence. Just before transduction, the culture media was removed and replaced by Opti-MEM I reduced Serum Medium (Gibco-Invitrogen, Life Technologies Inc., Texas, United States), to which the recombinant protein 10xHis-Xa_SITE_-TAT-α-globin-HA was then added. K-562 cells were treated with 50 μg ml^−1^ soluble protein for indicated intervals (30 min and 1 h), in order to evaluate its transduction efficiency.

RBCs samples were derived from either peripheral blood or bone marrow of HbH donor patients (Patients 1–5), and all subjects gave written informed consent. RBCs were, also, cultured in RPMI-1640 medium supplemented with 10% FBS and antibiotics/antimycotic at 37 °C, in 5% CO_2_. Just before transduction, the culture media was removed and replaced by Opti-MEM, to which the recombinant protein 10xHis-Xa_SITE_-TAT-α-globin-HA was then added (2 mg per 2 × 10^9^ RBCs) in the form of bacterial IBs, dissolved in 1 M l-Arg, solution (pH 8.0). RBCs were incubated for 4 h and 48 h. A solution of 1 M l-Arg, pH 8.0, was used as control treatment. For incubation times longer than 2 h, 1 × volume of RPMI-1640 medium (supplemented with FBS and antibiotics/antimycotic) was added.

At timed-intervals during incubation, both K-562 cells and RBCs cells from Patients 1 – 3 and Patient 5, were harvested and washed twice with PBS 1 × (pH 7.4) buffer and processed for Western Blot analysis. Briefly, cells were lysed in standard RIPA lysis buffer, supplemented with protease inhibitor cocktail (Roche, Basel, Switzerland) on ice for 15 min, centrifuged, collected as supernatants, and separated in a 14–15% SDS–polyacrylamide gel and immunoblotted, as described previously.

### Evaluation of the effects of transduction of recombinant fusion α-globin chain on the phenotype of HbH RBCs inclusion bodies (HbH-IBs)

Exposure of RBCs to supravital staining dyes (such as methylene blue, methylene violet or brilliant cresyl blue) provides a helpful tool for the screening of RBCs to detect potential HbH patient clinical phenotype, where β_4_ tetramers appear as precipitated inclusions (HbH-IBs) [27]. RBCs from five HbH patients were treated with bacterial-IBs, enriched in 10xHis-Xa_SITE_-TAT-α-globin-HA and washed twice with 1 × PBS. Subsequently, RBCs from four of them (Patients 1—4) were stained with methylene violet dye to evaluate the ratio of positively versus negative stained regarding the presence and/or absence of HbH-IBs, respectively.

HbH patients’ RBCs (more than 2 × 10^8^ cells in 500 μl) were incubated, in a RIA vial, for 30 min, at 37 °C, with 50 μl of 0.018 M NaNO_2_. Two drops of the mixture were placed in a new RIA vial and stained with two drops of a staining dye, containing methylene violet 2B and potassium oxalate monohydrate, for 30 min, at R/T. After slide coating, the slides were left to dry for 30 min at R/T and then examined under a light microscope. The percentage of positive cells was calculated by counting more than 1000 cells per assay and by dividing the number of positive cells by the number of total cells and multiplying by 100, while negative cells were calculated via the equation [1.00 – (Number of negative cells ÷ Number of total cells)] × 100.

### Statistical analysis

For the screening of HbH RBCs to evaluate the percentage of HbH-IBs, at least three independent biological repetitions from the four different HbH patients were performed. Statistical significance was achieved through an unpaired, parametric t test (*p* < 0.01 was the threshold for statistical significance) using Graph Pad Prism 6 (GraphPad Software San Diego, California, United States, http://www.graphpad.com).

## Results

### Cloning strategy and production of recombinant fusion proteins

**Fig. 1:**
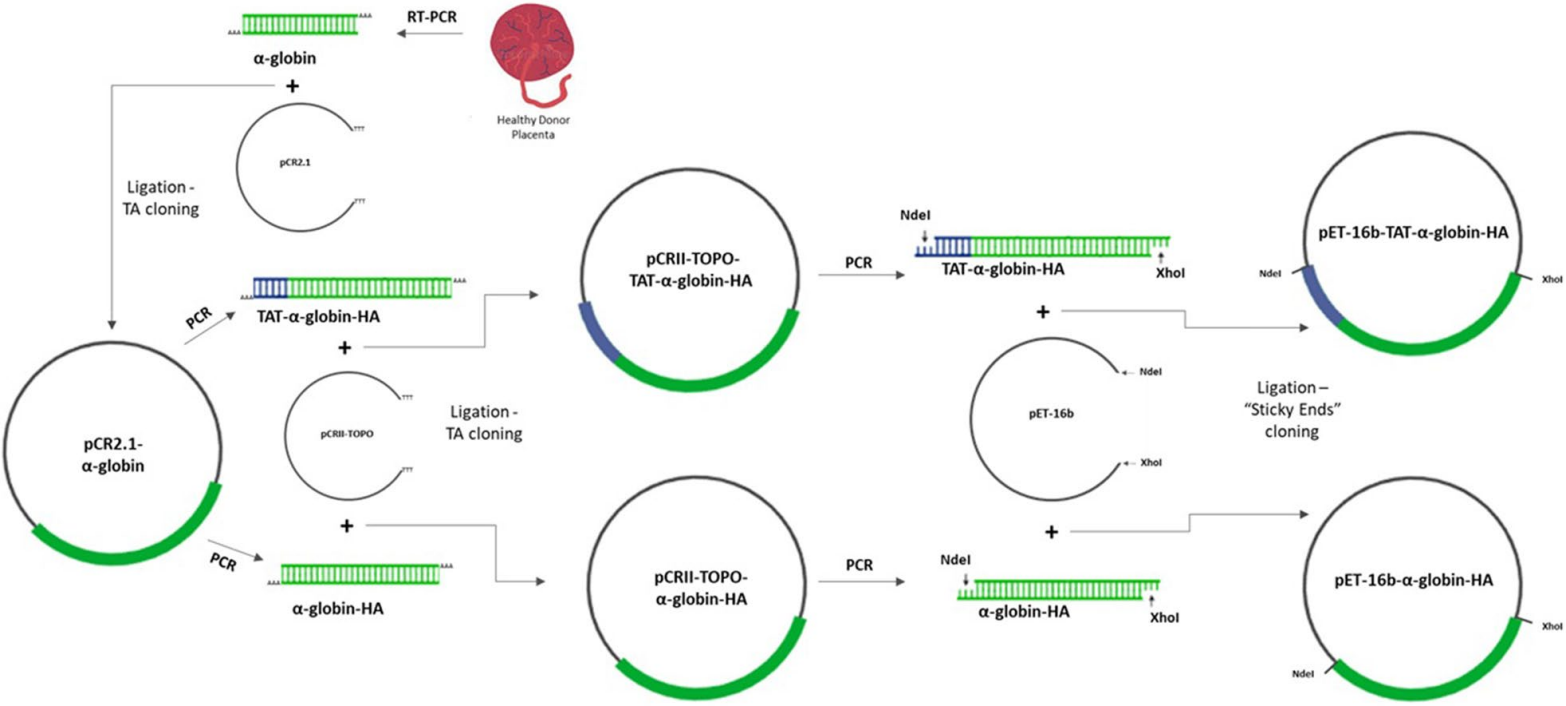
Schematic illustration of the cloning procedure, starting with RT-PCR from placenta tissue, derived from a healthy individual, followed by TA cloning of the a-globin CDS and then TA cloning of amplified TAT-α-globin-HA and α-globin-HA into pCRII-TOPO vector. Then, «sticky ends» cloning was conducted, with the restriction enzymes NdeI and XhoI, to generate the recombinant expression vectors pET-16b-TAT-α-globin-HA and pET-16b-α-globin-HA

**Fig. 2:**
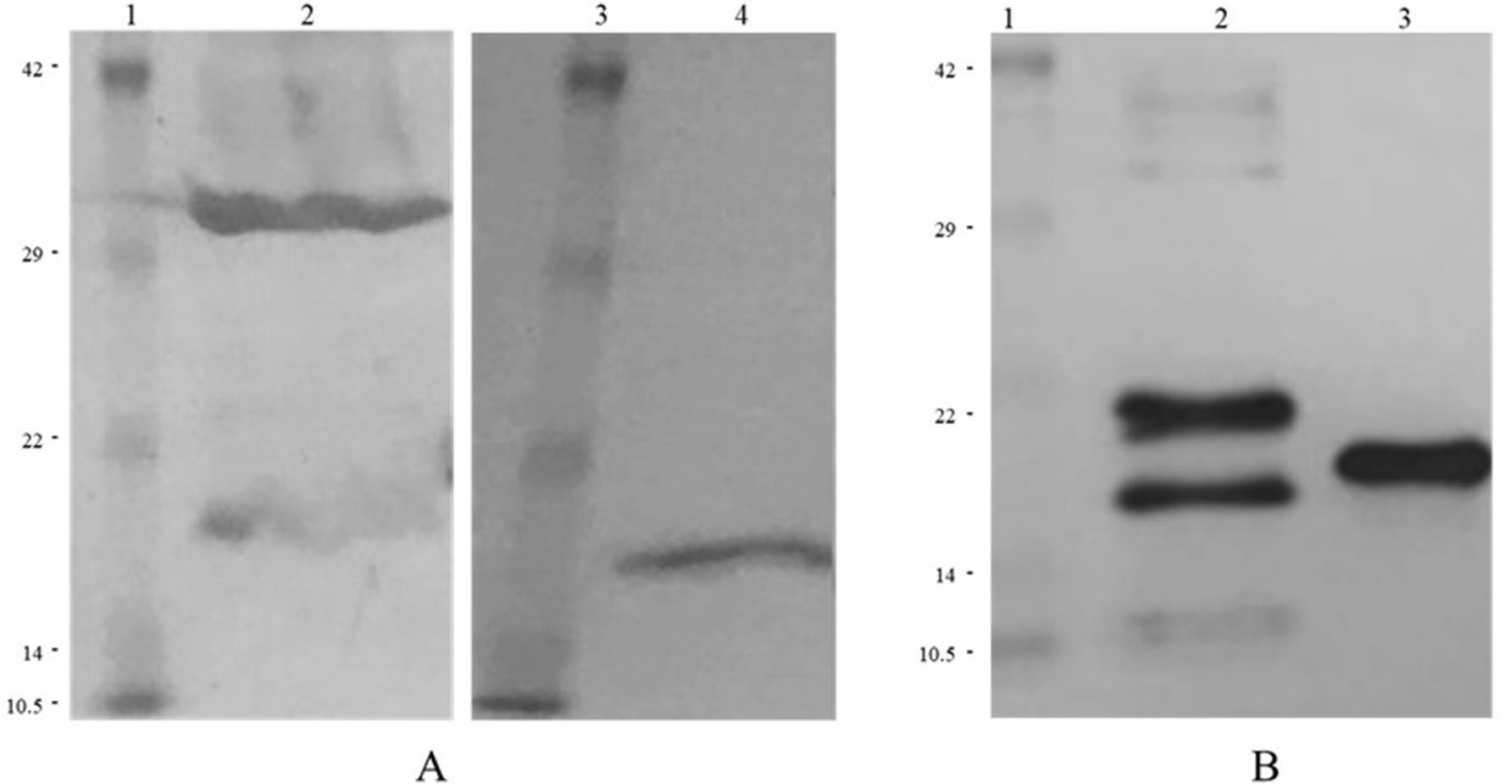
Western Blot of recombinant protein extracts, separated on 15% SDS-PAGE, transferred and immunoblotted with anti-His.IgG. **A** The bacterial-IBs’ protein extracts (in 1 M l-Arg, pH 8.0). Lane 1: protein molecular mass marker; Lane 2: 10xHis-Xa_SITE_-TAT-α-globin-HA (~ 20.2 kDa); Lane 3: protein molecular mass marker; Lane 4: 10xHis-Xa_SITE_-α-globin-HA (~ 18.7 kDa); **B** The soluble-purified by Ni^2+^-NTA chromatography-recombinant proteins, eluted with 10 mM imidazole. Lane 1: protein molecular mass marker; Lane 2: 10xHis-Xa_SITE_-TAT-α-globin-HA (~ 20.2 kDa); Lane 3: 10xHis-Xa_SITE_-α-globin-HA (~ 18.7 kDa). Higher bands at 29—41 kDa indicate the corresponding protein dimers, while lower bands may indicate proteolysis products

Three pCRII-TOPO-based and two pET-16b-based recombinant vectors were constructed for storage and high-level expression and purification, respectively, of heterologous TAT- and non-TAT fusion proteins in *E. coli* (Fig. 1 and Additional file 1: Fig. S1). The resultant purified 10xHis-Xa_SITE_-TAT-α-globin-ΗΑ was used for the study of protein transduction in RBCs (Fig. 2A).

Genetically modified bacteria, being transformed with pET-16b-ΤΑΤ-α-globin-ΗΑ vector and pET-16b-α-globin-ΗΑ vector, were induced by IPTG to produce recombinant 10xHis-Xa_SITE_-TAT-α-globin-ΗΑ (∼20.2 kDa) and 10xHis-Xa_SITE_-α-globin-ΗΑ (∼18.7 kDa) protein variants.

Purified bacterial-IBs, as previously described [17–19], successfully dissolved in 1 M l-Arg, pH 8.0, were analyzed by SDS − PAGE and Western Blot, and found to contain human recombinant 10xHis-Xa_SITE_-TAT-α-globin-ΗΑ and 10xHis-Xa_SITE_-α-globin-ΗΑ (Fig. 2A). No fusion protein was detected in bacterial-IBs derived from mock bacterial cells, transformed only with the pET-16b vector and induced by IPTG. Next, bacterial-IBs were filtered through Acrodisc32 mm Syringe Filter (with 0.45 μm Supor Membrane, Non-Pyrogenic, PALL Corp.) and kept at R/T.

**Fig. 3:**
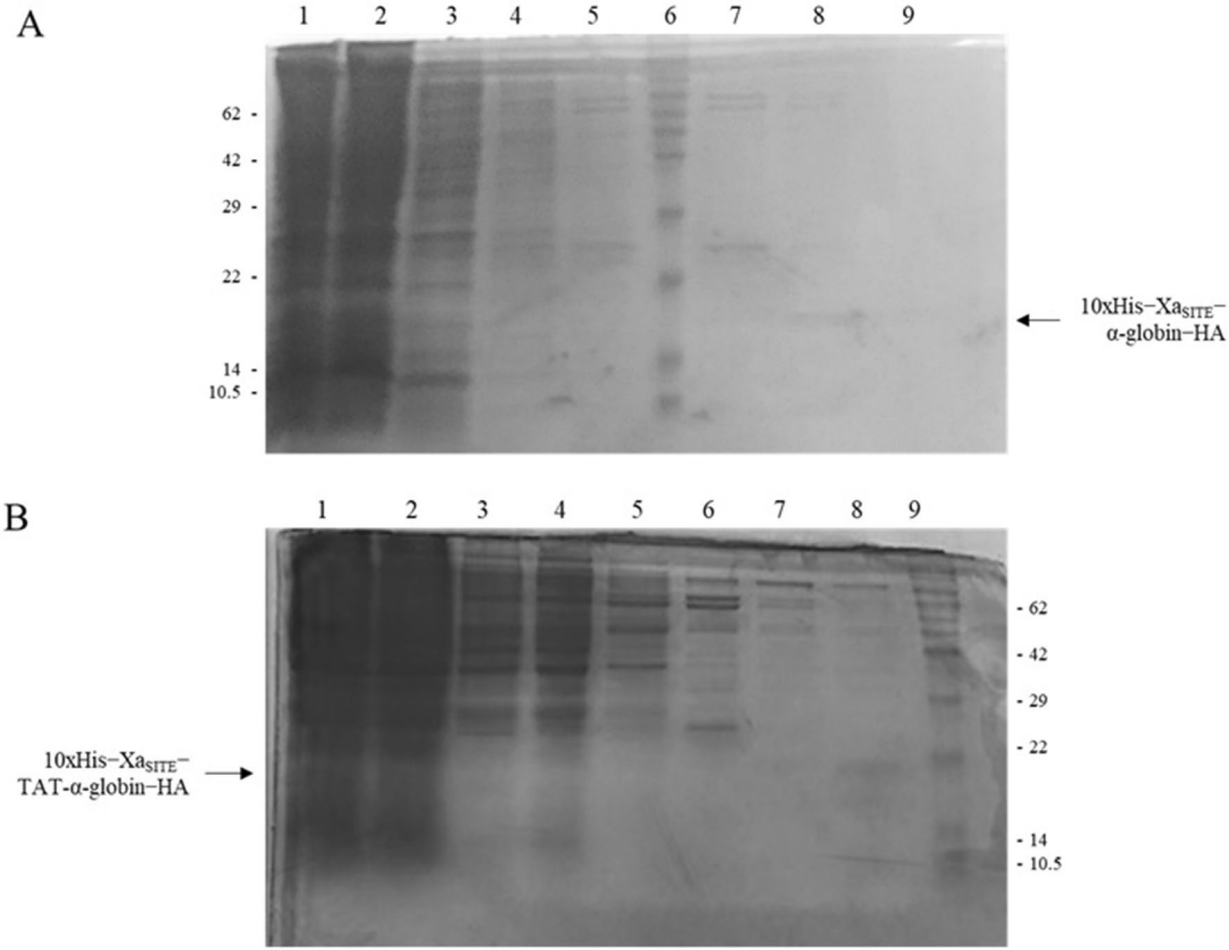
Retrieval of recombinant α-globin fusion protein variants purified by affinity Ni^2+^-NTA chromatography under denaturing conditions and analyzed by 15% SDS − PAGE and Coomassie Blue staining: **A** SDS-PAGE analysis of the purification of 10xHis-Xa_SITE_-α-globin-HA protein (~ 18.7 kDa). Lane 1: Crude extract of induced bacterial cells transformed by pET-16b-α globin-HA; Lane 2: Flow-through fraction of the Ni^2+^-NTA affinity column; Lane 3: Wash Buffer extract with 10 mM imidazole; Lane 4: Elution with 20 mM imidazole; Lane 5: Elution with 40 mM imidazole; Lane 6: protein molecular mass marker; Lane 7: Elution with 100 mM imidazole; Lane 8: Elution with 200 mM imidazole; Lane 9: Elution with 400 mM imidazole. **B** SDS-PAGE analysis of the purification of 10xHis-Xa_SITE_-TAT-α-globin-HA protein (~ 20.2 kDa). Lane 1: Crude extract of induced bacterial cells transformed by pET-16b-TAT-α globin-HA; Lane 2: Flow-through fraction of the Ni^2+^-NTA affinity column; Lane 3: Wash Buffer extract with 10 mM imidazole; Lane 4: Elution with 20 mM imidazole; Lane 5: Elution with 40 mM imidazole; Lane 6: Elution with 100 mM imidazole; Lane 7: Elution with 200 mM imidazole; Lane 8: Elution with 400 mM imidazole; Lane 9: protein molecular mass marker

Since the pET-16b-α-globin-HA contain a 10xHis tag, allowed the fusion protein to be purified by Ni^2+^-NTA chromatography and eluted from the column by adding gradient imidazole concertation buffers (containing 10 mM—400 mM imidazole) (Figs. 2B and 3). The eluted fusion protein was kept at 4 °C. The recombinant proteins 10xHis-Xa_SITE_-α-globin-ΗΑ (~ 18.7 kDa) and 10xHis-Xa_SITE_-TAT-β-globin-ΗΑ (∼20.6 kDa), both purified by Ni^2+^-NTA chromatography [19], were used for the in vitro assembly of α- and β- monomers, via size exclusion HPLC and the 10xHis-Xa_SITE_-TAT-α-globin-ΗΑ (~ 20.2 kDa) was used for the in vitro transduction of K-562 cells.

### Identification of produced fusion proteins by LC − MS/MS Technology for Protein Sequence

The bacterial-IBs, enriched in 10xHis-Xa_SITE_-TAT-α-globin-HA, as well as soluble (purified by Ni2 + -NTA chromatography) 10xHis-Xa_SITE_-α-globin-HA and 10xHis-Xa_SITE_-TAT-β-globin-HA, were analyzed by nanoLC − MS/MS analysis, which confirmed the presence of peptides for this protein.

**Fig. 4:**
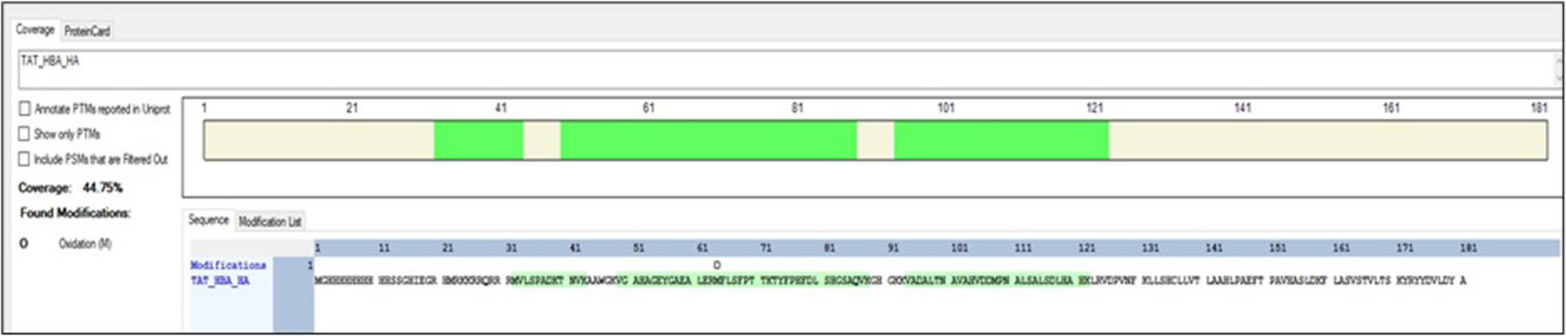
Identification of recombinant 10xHis-Xa_SITE_-TAT-α-globin-HA (TAT_HBA_HA) protein construct by LC − MS/MS. The highlighted peptides confidently identified cover 44.75% of the theoretical construct sequence

The results for the bacterial-IBs, enriched in 10xHis-Xa_SITE_-TAT-α-globin-HA confidently confirmed the overexpression of the alpha-globin protein (HBA1) with six tryptic peptides covering 44.75% of the construct sequence (Fig. 4). The bacterial-IBs extract also contained bacterial proteins, as expected. Identification details for the construct-specific peptides can be found in Additional file 1: Table S1.

### In vitro assembly of 10xHis-Xa_SITE_-α-globin-HA and 10xHis-Xa_SITE_-ΤΑΤ-β-globin-HA, analyzed by size exclusion HPLC

**Fig. 5:**
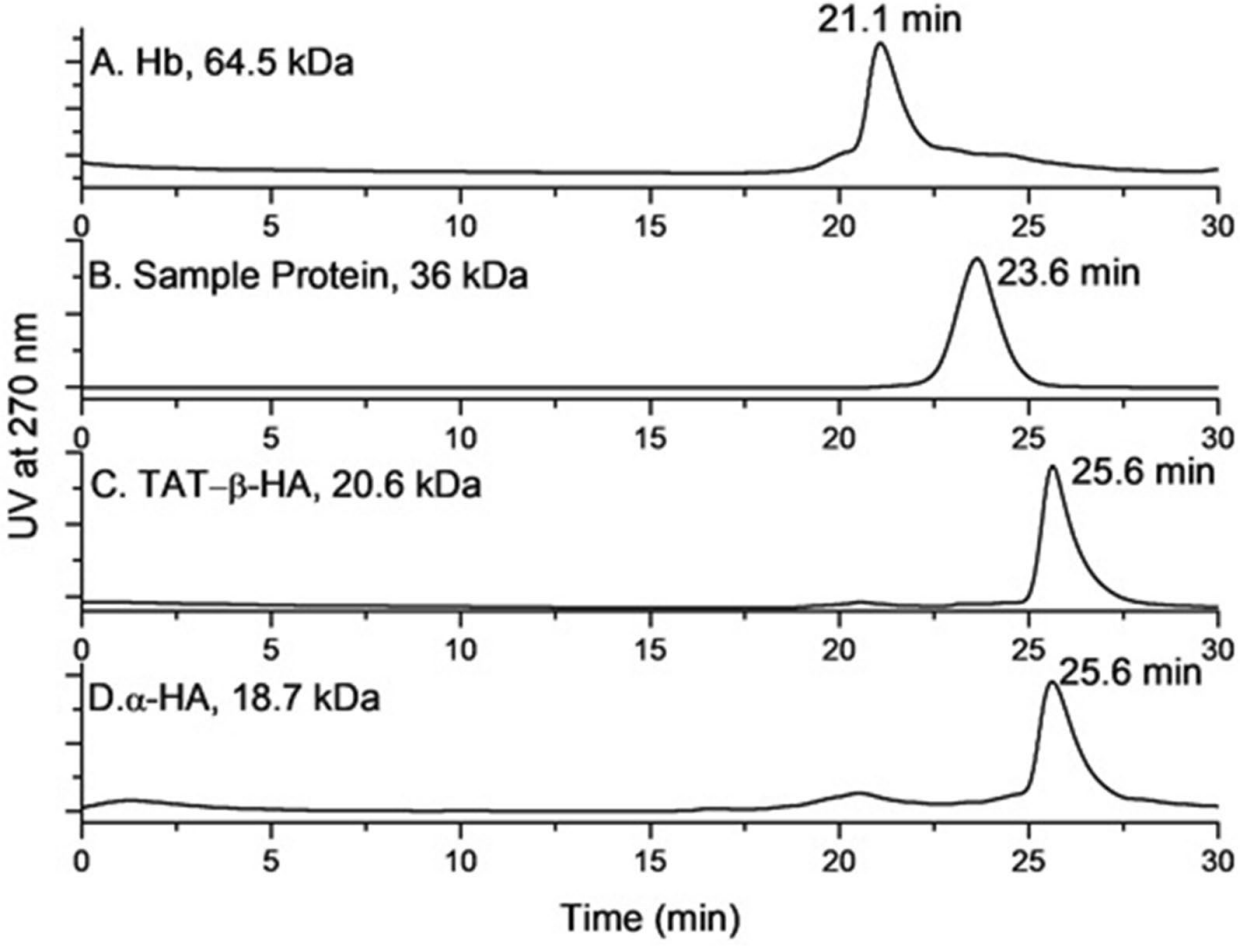
Size exclusion HPLC chromatograms: **A** hemoglobin tetramer (64.5 kDa) (t_R_ = 21.1 min); **B** soluble (in PBS 1 ×) sample protein (∼36 kDa) (t_R_ = 23.628 min); **C** soluble monomer 10xHis-Xa_SITE_-TAT-β-globin-HA (∼20.6 kDa), prior incubated with DTT for 48 h (t_R_ = 25.6 min); **D** soluble monomer 10xHis-Xa_SITE_-α-globin-HA (∼18.7 kDa), prior incubated with DTT for 48 h (t_R_ = 25.6 min)

From the SEC-HPLC analysis of the reaction mixtures between the soluble (purified by Ni^2+^-NTA chromatography) 10xHis-Xa_SITE_-α-globin-HA (α-) and 10xHis-Xa_SITE_-ΤΑΤ-β-globin-HA (β-) globin chains over 48 h at R/T, a new product-complex was formed with higher mass eluted at 23.7 min. Taking into consideration that: a) the bovine hemoglobin tetramer (64.5 kDa) elutes from the HPLC at 21.1 min (Fig. 5A), b) a recombinant protein-sample at 36 kDa elutes from the HPLC at 23.628 min (Fig. 5B), c) the 10xHis-Xa_SITE_-α-globin-HA and d) 10xHis-Xa_SITE_-ΤΑΤ-β-globin-HA, both eluted at 25.6 min (Fig. 5C and D), it seems that the peak at 23.7 min corresponds to the heterodimer 10xHis-Xa_SITE_-α-globin-HA / 10xHis-Xa_SITE_-ΤΑΤ-β-globin-HA (α- / β-) (prior incubated with DTT for reduction of globin-dimers [25]).

Proteomic analysis of the eluted HPLC peaks confidently identified the presence of 10xHis-Xa_SITE_-α-globin-HA in peak at 25.6 min (Fig. 5D) with 63% coverage (6 peptides) and 10xHis-Xa_SITE_-ΤΑΤ-β-globin-HA in peak at 25.6 min (Fig. 5C) with 61.9% (7 peptides), without detecting any other recombinant proteins in each different 25.6 min peak. The identified peptide specific details are summarized in Additional file 1: Tables S2 and S3.

**Fig. 6:**
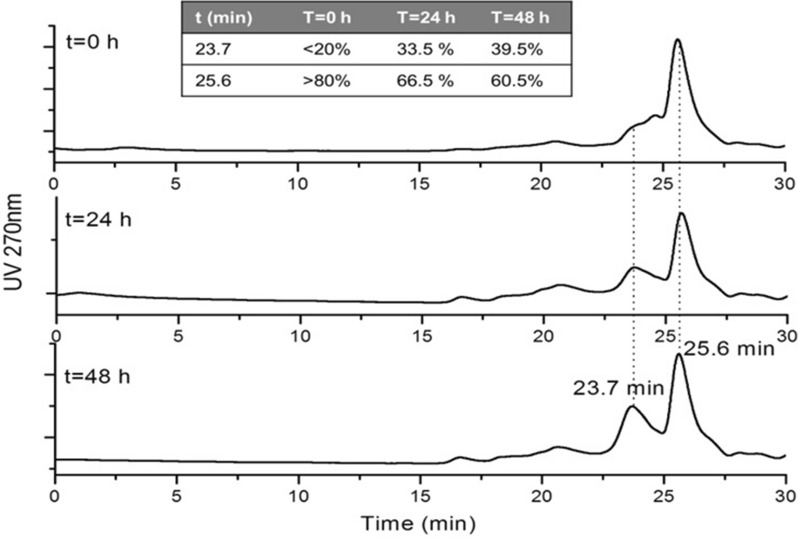
Size exclusion HPLC chromatograms of reaction mixtures of α- and β-monomers at t = 0 h, t = 24 h and t = 48 h

Furthermore, the integration of the HPLC peak at 23.7 min in relation to the peak at 25.6 min indicates an increase of the (α−/β−) heterodimer formed overtime (Fig. 6). Furthermore, incubation only of solely α- or β- monomers [also prior incubated with DTT for reduction of globin-dimers], over the same period (48 h) did not result in the formation of a dimer peak, as shown in Fig. 5C and D. This additionally indicates that a dimer (∼39.3 kDa) is formed only between α- and β- monomers [10xHis-Xa_SITE_-α-globin-HA (∼18.7 KDa) and 10xHis-Xa_SITE_-ΤΑΤ-β-globin-HA (∼20.6 kDa)], as shown in Fig. 6.

### Concentration- and time-dependent transduction of the 10xHis-Xa_SITE_-TAT-α-globin-ΗΑ protein in K-562 cells and in α-thalassemic patients’ RBCs

**Fig. 7:**
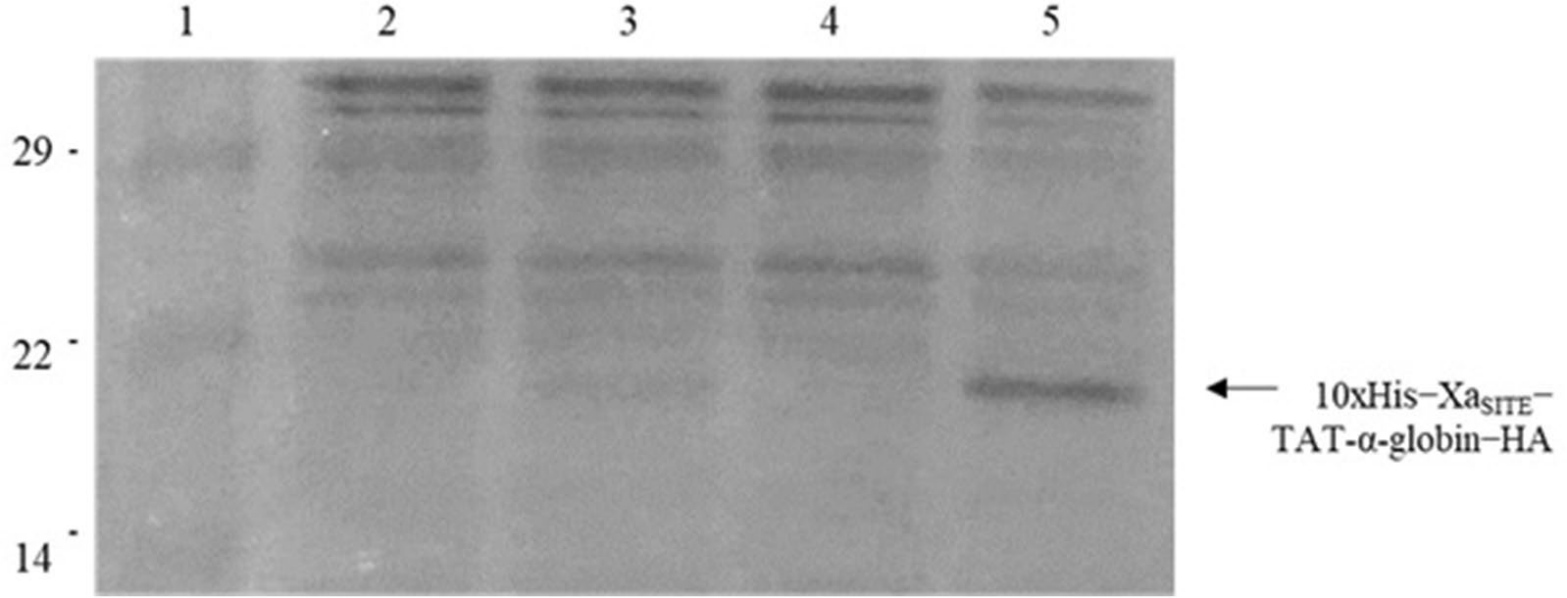
Western Blot of electrophoresed cell lysates derived from K-562 cells, transduced with 50 μg ml^−1^ soluble 10xHis − Xa_SITE_ − TAT − α-globin − HA, immunoblotted using anti-HA.IgG. Lane 1: protein molecular mass marker; Lane 2: lysate from control (untreated) cells, 30 min; Lane 3: lysate from cells transduced with 10xHis − Xa_SITE_ − TAT − α-globin − HA, incubated for 30 min.; Lane 4: lysate from control (untreated) cells, 1 h; Lane 5: lysate from cells transduced with 10xHis − Xa_SITE_ − TAT − α-globin − HA, incubated for 1 h

K-562 cells treatment was successfully carried out with 50 μg ml^−1^ of soluble and purified 10xHis-Xa_SITE_-TAT-α-globin-ΗΑ, for 30 min and 1 h under serum-free conditions (Opti-MEM I Reduced Serum Medium). Lysates derived from these cells were analyzed by SDS − PAGE and Western Blot, using the anti-HA.IgG. The 10xHis − Xa_SITE_ − TAT − α-globin − HA fusion protein was detected in the first hour of incubation in the cell lysates from the corresponding transduced K-562 cells and found to be at the expected molecular mass (∼20.2 kDa) (Fig. 7). The transduced 10xHis-Xa_SITE_-TAT-α-globin-ΗΑ did not seem to promote cytotoxicity (data not shown).

Bone marrow (2 × 10^6^ cells) or peripheral RBCs (2 × 10^9^ cells), derived from four individuals HbH patients, were successfully incubated with 2 mg bacterial-IBs (enriched in 10xHis-Xa_SITE_-TAT-α-globin-ΗΑ), dissolved in 1 M l-Arg, pH 8.0, for 24 h and 48 h, under reduced-serum conditions (Opti-MEM I Reduced Serum Medium). These results were confirmed by technical replicates and four independent experiments.

**Fig. 8:**
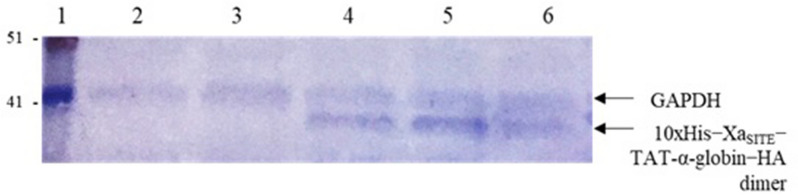
Western Blot of electrophoresed cell lysates derived from HbH patients’ peripheral RBCs (Patient 3), transduced with bacterial-IBs, enriched in 10xHis-Xa_SITE_-TAT-α-globin-HA, immunoblotted using anti-His.IgG and anti-GAPDH.IgG. Patient 3: Lane 1: protein molecular mass marker; Lane 2: lysate from control (untreated) cells, 48 h; Lane 3: lysate from cells incubated with 1 M l-Arg, for 48 h; Lanes 4–6: lysate from cells transduced with bacterial-IBs, enriched in 10xHis-Xa_SITE_-TAT-α-globin-HA, incubated for 48 h

Lysates derived from these cells were analyzed by SDS − PAGE and Western Blot, using the anti-His.IgG polyclonal antibody. The 10xHis-Xa_SITE_-TAT-α-globin-HA fusion protein was detected as a dimer (∼40 kDa), at 24 h and still at 48 h of incubation, in the cell lysates from the corresponding transduced patients’ RBCs (Fig. 8 and Additional file 1: Fig. S2).

### Reduction of HbH-IBs phenotype in α-thalassemic patients’ cells after treatment with 10xHis-Xa_SITE_-TAT-α-globin-HA

β_4_ aggregates appear as numerous dot size inclusions inside RBCs and are known as HbH-IBs [28]. Supravital stains, such as methylene violet, upon incubation, stain precipitated globin chains [28–30]. Every RBC sample, with the same number of cells, derived from individual HbH patients, untreated or incubated with 10xHis-Xa_SITE_-TAT-α-globin-HA for 48 h, was further incubated with methylene violet staining dye for an incubation period of 30 min and positive/negative cells, regarding HbH-IBs staining, were assessed.

**Fig. 9:**
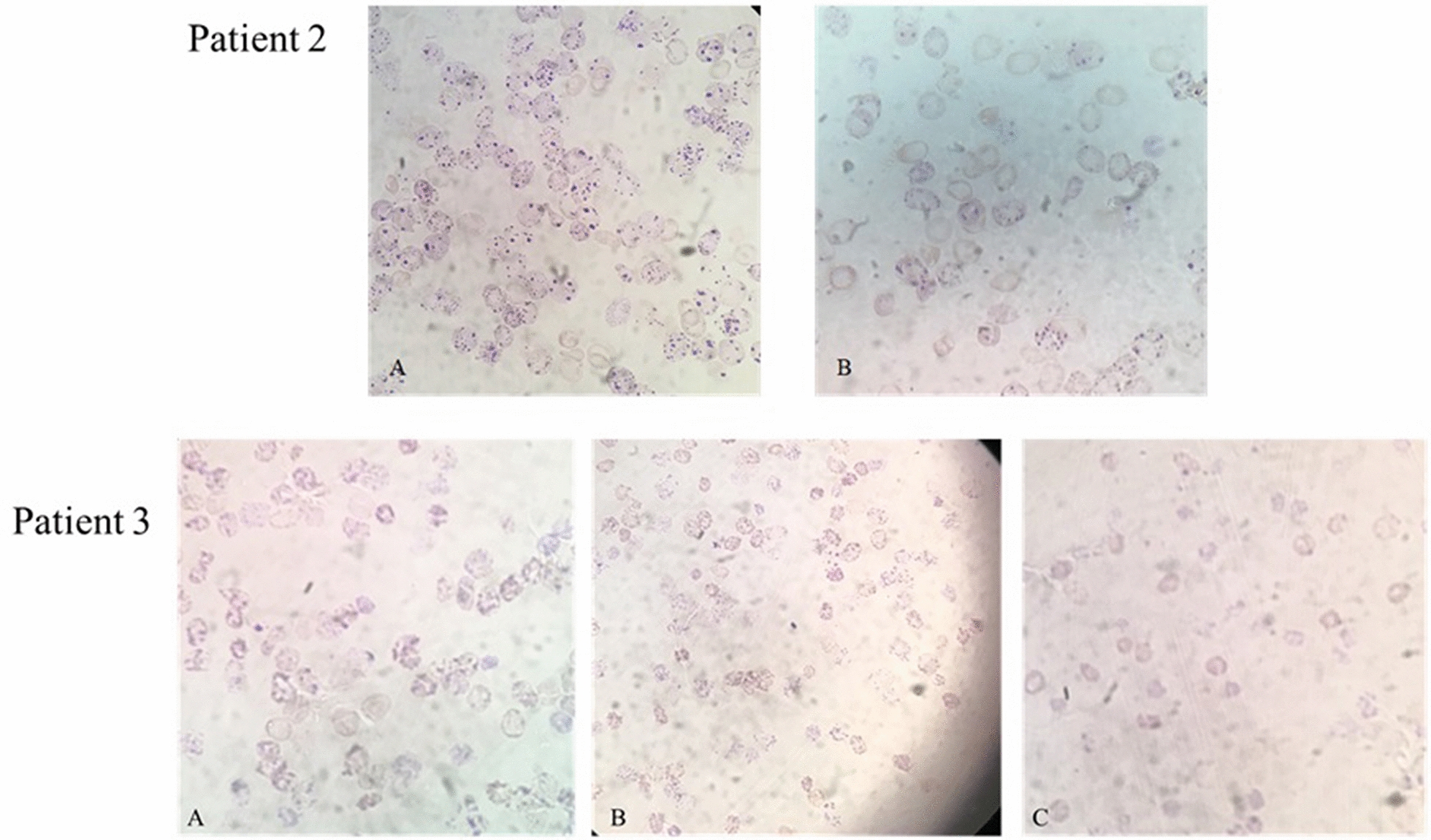
Methylene violet staining for the evaluation of HbH-IBs’ reduction, in HbH RBCs upon incubation with 10xHisXa_SITE_-TAT-α-globin-HA. Patient 2:** A** Untreated HbH patient’s RBCs, and **B** HbH patient’s RBCs incubated with IBs, enriched in 10xHis-Xa_SITE_-TAT-α-globin-HA, for 48 h. Patient 3: **A** Untreated HbH patient’s RBCs, **B** HbH patient’s RBCs incubated with 1 M l-Arg, for 48 h, **C** HbH patient’s RBCs incubated with IBs, enriched in 10xHis-Xa_SITE_-TAT-α-globin-HA, for 48 h

**Fig. 10:**
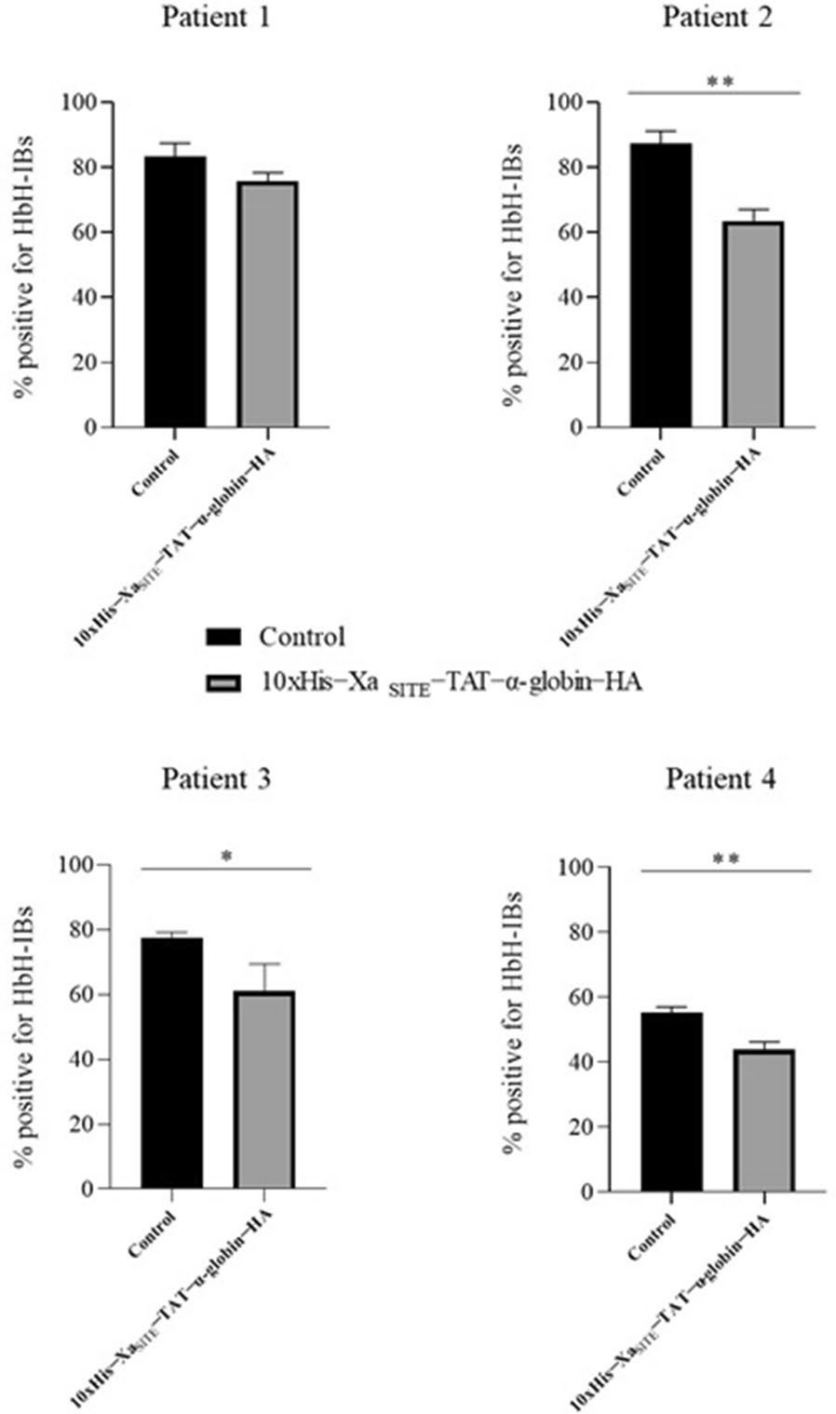
Diagrams for % positive for HbH-IBs in RBCs, derived from HbH patients (Patients 1–4), which were reduced after incubation with 10xHis-Xa_SITE_-TAT-α-globin-HA, for 48 h

All HbH patients were positive for HbH-IBs, with variable positive to negative percentage in untreated samples [Fig. 9, Patient 2 (A) and Patient 3 (A)]. HbH patients’ RBCs, that were incubated with solubilized in 1 M l-Arg ΙBs, enriched in 10xHis − Xa_SITE_-TAT-α-globin-HA, showed reduced staining [Fig. 9, Patient 2 (B) and Patient 3 (C)], after incubation with methylene violet, in comparison with the corresponding untreated samples. Furthermore, no significant reduction was observed after incubation with 1Μ l-Arg (ns) as a negative control [Fig. 9, Patient 3 (B)]. These results were confirmed by four independent experiments (Patient 1—4) and three technical replicates for Patients 2, 3, 4 and only two for Patient 1. Interestingly, the percentage of HbH-IBs varies among each patient (83.4%, 87.33%, 77.46% and 55.22% for Patients 1—4, respectively). The reduction percentage, after incubation with the 10xHis-Xa_SITE_-TAT-α-globin-HA, for 48 h—compared to respective untreated samples—varies from 7.65% in Patient 1, 16.45%, in Patient 3 (*p* = 0.0301 < 0.05) and 11.35% in Patient 4 (*p* = 0.0026 < 0.005), to the highest reduction percentage at 23.77% (*p* = 0.0013 < 0.005)*,* in Patient 2 (Fig. 10 and Additional file 1: Table S4).

## Discussion

Thalassemias, characterized by the absence or reduction of globin chain synthesis, are among the most prevalent genetic disorders worldwide, with 1.7% of the world’s population carrying thalassemic genes [31]. As far as α-thalassemia is concerned, carriers usually have no signs or symptoms, but people with the severe form as Hb Barts would need both intrauterine and lifelong transfusion [32].

As it has been also mentioned, HbH disease is caused by an absence or diminished synthesis of the α-globin chain of the hemoglobin molecule. Free alpha chains are insoluble and both γ- and β-chains make homotetramers (like β4), which are unstable, and may precipitate within the cell, leading to a variety of clinical manifestations [33] (aggregation within RBCs, forming HbH-IBs and resulting in hemolysis) [34].

In developed countries, treatment of thalassemias is also still far from ideal, requiring lifelong transfusion or allogeneic bone marrow transplantation. Gene therapy has made noteworthy, albeit slow, progress and is being tested in clinical trials [35–37]. Furthermore, novel genetic therapeutic approaches for modulating the severity of thalassemias, like gene editing, are in progress [38]. Hence, there is still an unmet need for new, alternative, and effective therapeutic strategies for treatment of this life-limiting disease to render patients be transfusion-independent and able to live a normal life.

Novel therapeutic approaches for severe HbH syndromes would include PRT. The PTD technology, towards PRT, has already been successfully applied by our group in vitro, as a therapeutic approach for β-thalassemia, producing human recombinant β-globin chain variants in fusion with TAT [19].

In this study, we produced the human recombinant α-globin chain, fused to TAT (the most used PTD), in bacteria, to be intracellularly transduced into cells of HbH patients. We cloned and produced the human recombinant 10xHis-Xa_SITE_-α-globin-HA and investigated the ability of human recombinant α- and *β-*globin chain variants to form α-/β- complexes in vitro. The recombinant proteins were solely, prior, incubated with DTT for reduction of globin-dimer formation and the corresponding monomer size exclusion HPLC peaks were identified by mass spectra molecular analysis, confirming the presence of each monomer. When these monomers (10xHis-Xa_SITE_-α-globin-HA and 10xHis-Xa_SITE_-TAT-β-globin-HA) were incubated together, α-/β- heterodimers were formed overtime (48 h).

For the preliminary evaluation of the transduction efficiency of the recombinant TAT-fused α-globin, in vitro transduction experiments were conducted using human K-562 cells. Although, K-562 cell-line is a suitable model system for studying β-thalassemia (absence of production of mature β-globin nascent chains) [19] and α-globin chains are produced normally, this cell-line was only used to assess the transducibility of the 10xHis-Xa_SITE_-α-globin-HA, since bearing His- and HA- tags would permit its distinction from the endogenous α-chain. The successful transduction in K-562 was shown from the first hour of incubation, thus we proceeded in the transduction experiments of the HbH patients’ RBCs.

Mature RBCs are known for their incapability to produce globin chains. In the case of HbH RBCs, the excess of β-globin chains, due to the absence of the α-globin chains, leads to the formation of the β_4_ aggregates, known to be unstable, with a high oxygen affinity and thus unable to effectively deliver oxygen, leading to hemolytic anemia [39]. The heterogeneity characterizing the molecular basis of α-thalassemia (deletions or point mutations in the α-globin genes), affecting the expression of the duplicated α-genes, leads to a remarkable clinical variation among α-thalassemic patients [40], reflected also in the amount of β_4_ aggregates formed. Remarkably, the number HbH-IBs (containing the β_4_ tetramers)—observed during the incubation assay with methylene violet, in order to stain precipitated globin chains—varied among different patients analyzed, in correspondence to any genetic profile variation.

As a therapeutic perspective, a potential dissociation of the β_4_ aggregates and their reduction may lead to a smoother disease phenotype and reduction of their harmful effects. In this study, 10xHis-Xa_SITE_-TAT-α-globin-HA, derived from bacterial-IBs and identified by mass spectra molecular analysis, was successfully transduced into HbH patients’ RBCs and revealed a potential phenotype complementation, since HbH-IBs were found statistically significant reduced, upon incubation with methylene violet staining. The percentages of the HbH-IBs reduction also varies between different α-thalassemic patients, pointing out the heterogeneity of the clinical phenotype and the potential effect on the response of each patient to such treatment, indicating the need for a more pharmacogenomic approach.

Whether the results of this study propose the potential disassociation of β_4_ aggregates, probably via a tendency of globin chains to form bonds [40] and subsequent formation of α-/β- heterodimers, thus reducing the HbH-IBs, needs further investigation. However, it seems that this significant reduction of the β_4_ aggregates (HbH-IBs) could provide patients with a therapeutic advantage, by preventing the most severe clinical phenotypes with increased peripheral hemolysis and ineffective erythropoiesis in such patients.

## Supplementary Information

**Additional file 1.** Additional methodology, figures and tables.

## Data Availability

Not applicable.

## Abbreviations

Bacterial-IBs: Bacterial inclusion bodies
BHFS: Hb Bart's hydrops fetalis
CDS: Coding sequence
CPPs: Cell penetrating peptides
FBS: Fetal bovine serum
HA: Hemagglutinin
HbH: Hemoglobin H
HbH-IBs: HbH RBCs inclusion bodies
l-Arg: l-Arginine
PRT: Protein replacement therapy
PTDs: Protein transduction domains
R/T: Room temperature
RBC: Red blood cells
SEC: Size exclusion chromatography
Soluble: Purified by Ni^2^^+^-NTA chromatography
