# Supplementary material for: PTD-mediated delivery of α-globin chain into Κ-562 erythroleukemia cells and α-thalassemic (HBH) patients’ RBCs ex vivo in the frame of Protein Replacement Therapy
Source: J Biol Res (Thessalon). 2021 Jul 20;28:16. doi: 10.1186/s40709-021-00148-3 (PMC8290593; doi:10.1186/s40709-021-00148-3)
Supplement: Supplementary file 1 — Additional file 1. Additional methodology, figures and tables. [file 40709_2021_148_MOESM1_ESM.docx]

**Additional Information**

**Additional Methods**

LC−MS/MS technology for recombinant protein identification

a) Tryptic Digestion

All recombinant proteins were digested with trypsin using the single-pot, solid-phase-enhanced sample-preparation (SP3) technology digestion protocol (1) and the extracted peptide solution was dried down in a vacuum centrifuge (Savant). The samples were reconstituted with 30 μL of 2% (v/v) acetonitrile/0.1% (v/v) formic acid solution, sonicated in a water bath for 3 min, and analyzed with nanoLC−MS/MS.

b) LC−MS/MS analysis

The purified peptides were analyzed by nanoHPLC−MS/MS, using an QE Orbitrap HF-X (Thermo Fisher Scientific, Waltham, MA, U.S.A.), equipped with a nanospray source. The peptide mixtures (10 μL) were preconcentrated at a flow rate of 15 μL/min for 2 min using a C18 trap column (Acclaim PepMap) and then loaded onto a 50 cm C18 column (75 μm ID, particle size 2 μm, 100 Å, Acclaim PepMap RSLC, Thermo Scientific). The binary pumps of the HPLC (RSLCnano, Thermo Fisher Scientific) contained solution A (2% (v/v) ACN in 0.1% (v/v) formic acid) and solution B (80% ACN in 0.1% formic acid). The peptides were separated using a linear gradient of 4−30% B in 68 min at a flow rate of 300 nL/min. The column was placed in an oven operating at 50 °C. Full-scan MS spectra were acquired in the Orbitrap (m/z 390−1400) in profile mode and data-dependent acquisition with the resolution set to 60,000 at m/z 400 and the automatic gain control target at 3 x10^6^. The fifteen most intense ions were sequentially isolated, and HCD was used to generate fragments detected in the Orbitrap with 15,000 resolving power. Dynamic exclusion was set to 60 s. Ions with single charge states were excluded. A lock mass of m/z 445.120025 was used for internal calibration. The Thermo Scientific™ Xcalibur™ software was used to control the system and acquire the raw files.

c) Data Processing

Mass spectra were searched using Proteome Discoverer 2.4 (Thermo Fisher Scientific) using the Sequest HT search algorithm. The Uniprot reviewed FASTA (298876 entries, 10/2019) database was used in this search, the precursor mass range was set to 350-3000 Da, the mass error tolerance was set to 20 ppm, and the fragment mass error tolerance to 0.5 Da. Enzyme specificity was set to trypsin, carbamidomethylation of cysteines (57.021) was set as variable modifications, oxidation of methionines (+15.995) and acetylation of protein N-terminus (+42.011) was considered as variable modifications. Fixed value PSM validator was used with default settings.

1. Wisniewski JR, Zougman A, Nagaraj N, Mann M. Universal sample preparation method for proteome analysis. Nat Methods. 2009;6(5):359-62. doi. 10.1038/nmeth.1322.

**Additional Figures**

**Additional Figure S1**. *Generation of the recombinant plasmid vectors for* *storage and for* *high-level expression and purification of TAT- and non-TAT fusion proteins in E. coli. The maps of the plasmids A. pCR2.1-α-globin, B. pCRII-TOPO-α-globin-HA, C. pCRII-TOPO-TAT-α-globin-HA****,*** *D. pET16b-10xHis-Xa_SITE_-α-globin-HA and E. pET16b-10xHis-Xa_SITE_-TAT-α-globin-HA.*


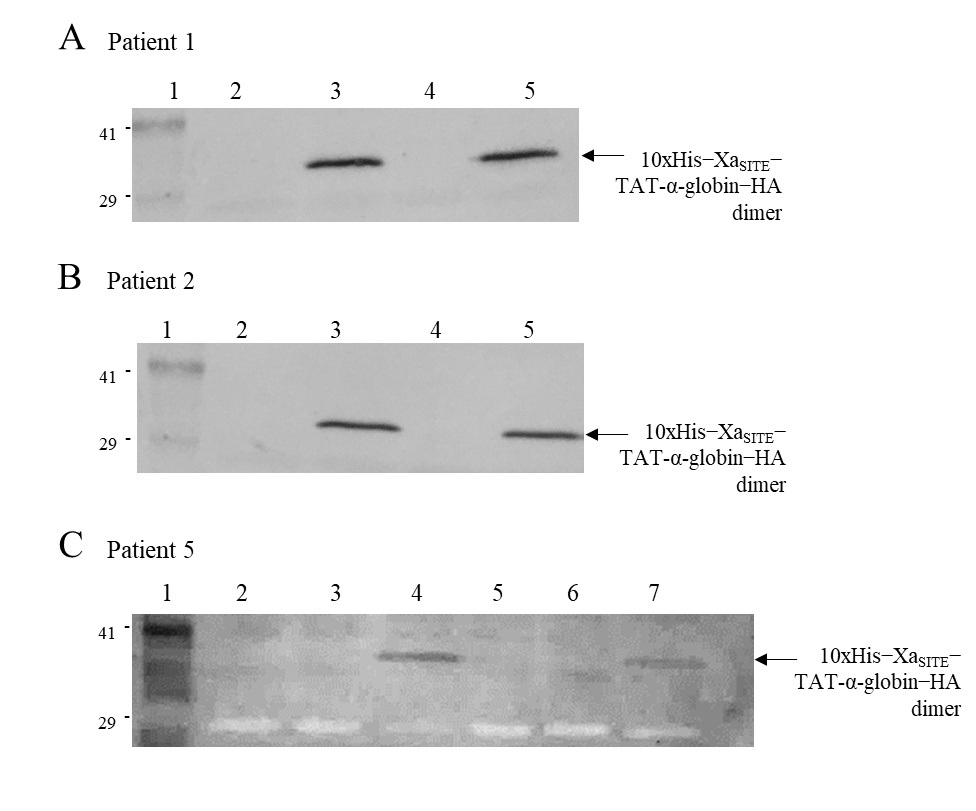


**Additional Figure S2**. *Western blotting of electrophoresed cell lysates derived from HbH patients’ peripheral RBCs (****Α****. Patient 1 and* ***Β****. Patient 2) or from bone marrow derived RBCs (****C****. Patient 5), transduced with bacterial-IBs, enriched in 10xHis-Xa_SITE_-TAT-α-globin-HA and immunoblotted using anti-His.IgG.*

***A.*** *Patient 1: Lane 1: protein molecular mass marker; Lane 2: lysate from control (untreated) cells, 24 h; Lane 3: lysate from cells transduced with bacterial-IBs, enriched in 10xHis-Xa_SITE_-TAT-α-globin-HA, incubated for 24 h; Lane 4: lysate from control (untreated) cells, 48 h; Lane 5: lysate from cells transduced with bacterial-IBs, enriched in 10xHis-Xa_SITE_-TAT-α-globin-HA, incubated for 48 h.*

***B.*** *Patient 2: Lane 1: protein molecular mass marker; Lane 2: lysate from control (untreated) cells, 24 h; Lane 3: lysate from cells transduced with bacterial-IBs, enriched in 10xHis-Xa_SITE_-TAT-α-globin-HA, incubated for 24 h; Lane 4: lysate from control (untreated) cells, 48 h; Lane 5: lysate from cells transduced with bacterial-IBs, enriched in 10xHis-Xa_SITE_-TAT-α-globin-HA, incubated for 48 h; Lane 6: IBs, enriched in 10xHis-Xa_SITE_-TAT-α-globin−HA;*

***C.*** *Patient 5: Lane 1: protein molecular mass marker; Lane 2: lysate from control (untreated) cells, 24 h; Lane 3: lysate from cells incubated with 1 M l-Arg, for 24 h; Lane 4: lysate from cells transduced with bacterial-IBs, enriched in 10xHis-Xa_SITE_-TAT-α-globin-HA, incubated for 24 h; Lane 5: lysate from control (untreated) cells, 48 h; Lane 6: lysate from cells incubated with 1 M l-Arg, for 48 h; Lane 7: lysate from cells transduced with bacterial-IBs, enriched in 10xHis-Xa_SITE_-TAT-α-globin-HA, incubated for 48 h.*

**Supplementary Tables**

**Additional Table S1.** The characteristics of the tryptic HBA1 specific peptides identified from the bacterial-IBs, enriched 10xHis-Xa_SITE_-TAT-α-globin-HA.

| **Sequence** | **Modifications** | **Qvality PEP** | **Qvality q-value** | **# Protein Groups** | **# Proteins** | **# PSMs** | **Master Protein Accessions** | **# Missed Cleavages** | **Theo. MH+ [Da]** | **Confidence (by Search Engine)** | **Percolator q-Value (by Search Engine)** | **Percolator PEP (by Search Engine)** | **XCorr (by Search Engine)** |
| --- | --- | --- | --- | --- | --- | --- | --- | --- | --- | --- | --- | --- | --- |
| VADALTNAVAHVDDMPNALSALSDLHAHK | 2.5e-6 | 2.5e-6 | 8.0e-4 | 1 | 8 | 3 | P69905 | 0 | 2996.48944 | High | 0.0007321 | 4.68E-07 | 5.03 |
| VGAHAGEYGAEALER | 2.6e-6 | 2.6e-6 | 8.0e-4 | 1 | 3 | 3 | P69905 | 0 | 1529.73430 | High | 0.0007321 | 4.827E-07 | 4.05 |
| MVLSPADKTNVK | 1.1e-3 | 1.1e-3 | 8.0e-4 | 2 | 12 | 1 | P69905; P01928 | 1 | 1302.70860 | High | 0.0007321 | 0.0004241 | 3.63 |
| MFLSFPTTK | 3.1e-2 | 3.1e-2 | 1.5e-3 | 7 | 80 | 5 | P69905; P01972; P09420; P19002; P63107; P07414; P01928 | 0 | 1071.55433 | High | 0.001199 | 0.01821 | 1.78 |
| MFLSFPTTK | 1×Oxidation [M1] | 1.4e-2 | 8.0e-4 | 7 | 80 | 4 | P69905; P01972; P09420; P19002; P63107; P07414; P01928 | 0 | 1087.54924 | High | 0.0007321 | 0.007544 | 1.78 |
| TYFPHFDLSHGSAQVK | 4.4e-6 | 4.4e-6 | 8.0e-4 | 5 | 72 | 7 | P69905; P07425; P19002; P63107; P01928 | 0 | 1833.89186 | High | 0.0007321 | 8.855E-07 | 3.08 |

**Additional Table S2.** The characteristics of the tryptic HBA1 specific peptides identified from soluble (purified by Ni^2+^-NTA chromatography) 10xHis-Xa_SITE_-α-globin-HA.

| **Checked** | **Master** | **Accession** | **Description** | **Coverage [%]** | **# Peptides** | **# PSMs** | **# Unique Peptides** | **# AAs** | **MW [kDa]** | **calc. pI** | **Score Sequest HT: Sequest HT** | **# Peptides (by Search Engine): Sequest HT** | **# Protein Groups** | **Score Sequest HT: Sequest HT** | **# Peptides (by Search Engine): Sequest HT** | **# Protein Groups** |
| --- | --- | --- | --- | --- | --- | --- | --- | --- | --- | --- | --- | --- | --- | --- | --- | --- |
| TRUE | Master Protein | P69905 | Hemoglobin subunit alpha OS=Homo sapiens OX=9606 GN=HBA1 PE=1 SV=2 | 63 | 6 | 37 | 6 | 142 | 15,2 | 8,68 | 119,59 | 6 | 1 | 51,21 | 4 | 1 |

| **Checked** | **Confidence** | **Sequence** | **Modifications** | **Qvality PEP** | **Qvality q-value** | **# Protein Groups** | **# Proteins** | **# PSMs** | **Master Protein Accessions** | **# Missed Cleavages** | **Theo. MH+ [Da]** | **Confidence (by Search Engine): Sequest HT** | **Percolator q-Value (by Search Engine): Sequest HT** | **Percolator PEP (by Search Engine): Sequest HT** | **XCorr (by Search Engine): Sequest HT** |
| --- | --- | --- | --- | --- | --- | --- | --- | --- | --- | --- | --- | --- | --- | --- | --- |
| FALSE | High | FLASVSTVLTSK |  | 9,56E-05 | 0,000515 | 5 | 58 | 3 | P69905; B3EWE3; P07421; P01929; P01935 | 0 | 1252,715 | High | 0,000371 | 2,67E-05 | 2,21 |
| FALSE | High | MFLSFPTTK |  | 0,010183 | 0,000858 | 4 | 79 | 4 | P69905; P07421; P01929; P01935 | 0 | 1071,554 | High | 0,000529 | 0,004429 | 1,56 |
| FALSE | High | MFLSFPTTK | 1xOxidation [M1] | 0,009663 | 0,000858 | 4 | 79 | 10 | P69905; P07421; P01929; P01935 | 0 | 1087,549 | High | 0,000529 | 0,004181 | 1,9 |
| FALSE | High | TYFPHFDLSHGSAQVK |  | 7,72E-07 | 0,000515 | 4 | 72 | 8 | P69905; P01973; P07421; P01929 | 0 | 1833,892 | High | 0,000371 | 1,37E-07 | 3,54 |
| FALSE | High | VADALTNAVAHVDDMPNALSALSDLHAHK |  | 1,92E-09 | 0,000515 | 1 | 7 | 3 | P69905 | 0 | 2996,489 | High | 0,000371 | 1,93E-10 | 4 |
| FALSE | High | VADALTNAVAHVDDMPNALSALSDLHAHK | 1xOxidation [M15] | 1,88E-05 | 0,000515 | 1 | 7 | 1 | P69905 | 0 | 3012,484 | High | 0,000371 | 4,49E-06 | 2,85 |

**Additional Table S3.** The characteristics of the tryptic HBΒ specific peptides identified from soluble (purified by Ni^2+^-NTA chromatography) 10xHis-Xa_SITE_-TAT-β-globin-HA.

| **Checked** | **Master** | **Accession** | **Description** | **Coverage [%]** | **# Peptides** | **# PSMs** | **# Unique Peptides** | **# AAs** | **MW [kDa]** | **calc. pI** | **Score Sequest HT: Sequest HT** | **# Peptides (by Search Engine): Sequest HT** | **# Protein Groups** | **# Protein Groups** |
| --- | --- | --- | --- | --- | --- | --- | --- | --- | --- | --- | --- | --- | --- | --- |
| TRUE | Master Protein | P68871 | Hemoglobin subunit beta OS=Homo sapiens OX=9606 GN=HBB PE=1 SV=2 | 61.9 | 7 | 74 | 7 | 147 | 16 | 7.28 | 221.82 | 7 | 1 | 1 |

| **Checked** | **Confidence** | **Sequence** | **Modifications** | **# Protein Groups** | **# Proteins** | **# PSMs** | **Master Protein Accessions** | **# Missed Cleavages** | **Theo. MH+ [Da]** | **Confidence (by Search Engine): Sequest HT** | **XCorr (by Search Engine): Sequest HT** |
| --- | --- | --- | --- | --- | --- | --- | --- | --- | --- | --- | --- |
| TRUE | High | EFTPPVQAAYQK |  | 1 | 1 | 2 | P68871 | 0 | 1378,7 | High | 2,73 |
| TRUE | High | FFESFGDLSTPDAVMGNPK |  | 1 | 3 | 11 | P68871 | 0 | 2058,948 | High | 4,97 |
| TRUE | High | FFESFGDLSTPDAVMGNPK | 1xOxidation [M15] | 1 | 3 | 14 | P68871 | 0 | 2074,943 | High | 3,85 |
| TRUE | High | LLVVYPWTQR |  | 1 | 11 | 3 | P68871 | 0 | 1274,726 | High | 2,74 |
| TRUE | High | SAVTALWGK |  | 1 | 4 | 9 | P68871 | 0 | 932,52 | High | 2,65 |
| TRUE | High | VLGAFSDGLAHLDNLK |  | 1 | 6 | 16 | P68871 | 0 | 1669,891 | High | 5,15 |
| TRUE | High | VNVDEVGGEALGR |  | 1 | 3 | 12 | P68871 | 0 | 1314,665 | High | 4,06 |
| TRUE | High | VVAGVANALAHK |  | 1 | 2 | 7 | P68871 | 0 | 1149,674 | High | 4,01 |

**Additional Table S4.** *% positive for HbH-IBs in RBCs, derived from HbH Patients 1 - 4, after incubation with 10xHis-Xa_SITE_-TAT-α-globin-HA, for 48 h.*

| **HbH Patient** | **n** | **% HbH-IBs** | | | |
| --- | --- | --- | --- | --- | --- |
|  |  | **Untreated** | **Incubation with**  **10xHis-Xa_SITE_-TAT-α-globin-HA** | **Reduction in HbH-IBs** | ***P*-value** |
| 1 | 2 | 83.4 | 75.75 | 7.65 | - |
| 2 | 3 | 87.3 | 63.53 | 23.77 | 0.0013** |
| 3 | 3 | 77.46 | 61.01 | 16.45 | 0.0301* |
| 4 | 3 | 55.22 | 43.87 | 11.35 | 0.0026** |
